# Supplementary material for: Mutations in PCYT2 disrupt etherlipid biosynthesis and cause a complex hereditary spastic paraplegia
Source: Brain. 2019 Oct 22;142(11):3382–97. doi: 10.1093/brain/awz291 (PMC6821184; doi:10.1093/brain/awz291)
Supplement: awz291_Supplementary_Data [file awz291_supplementary_data.zip › awz291-Suppl_data/Supplementary_Data4.zip!/Supplementary information 4 - plasma lipidomics.zip!/Plasma lipidomics/Control vs Patient1/Statistical plots and data/LPA species/Boxplots.pdf]

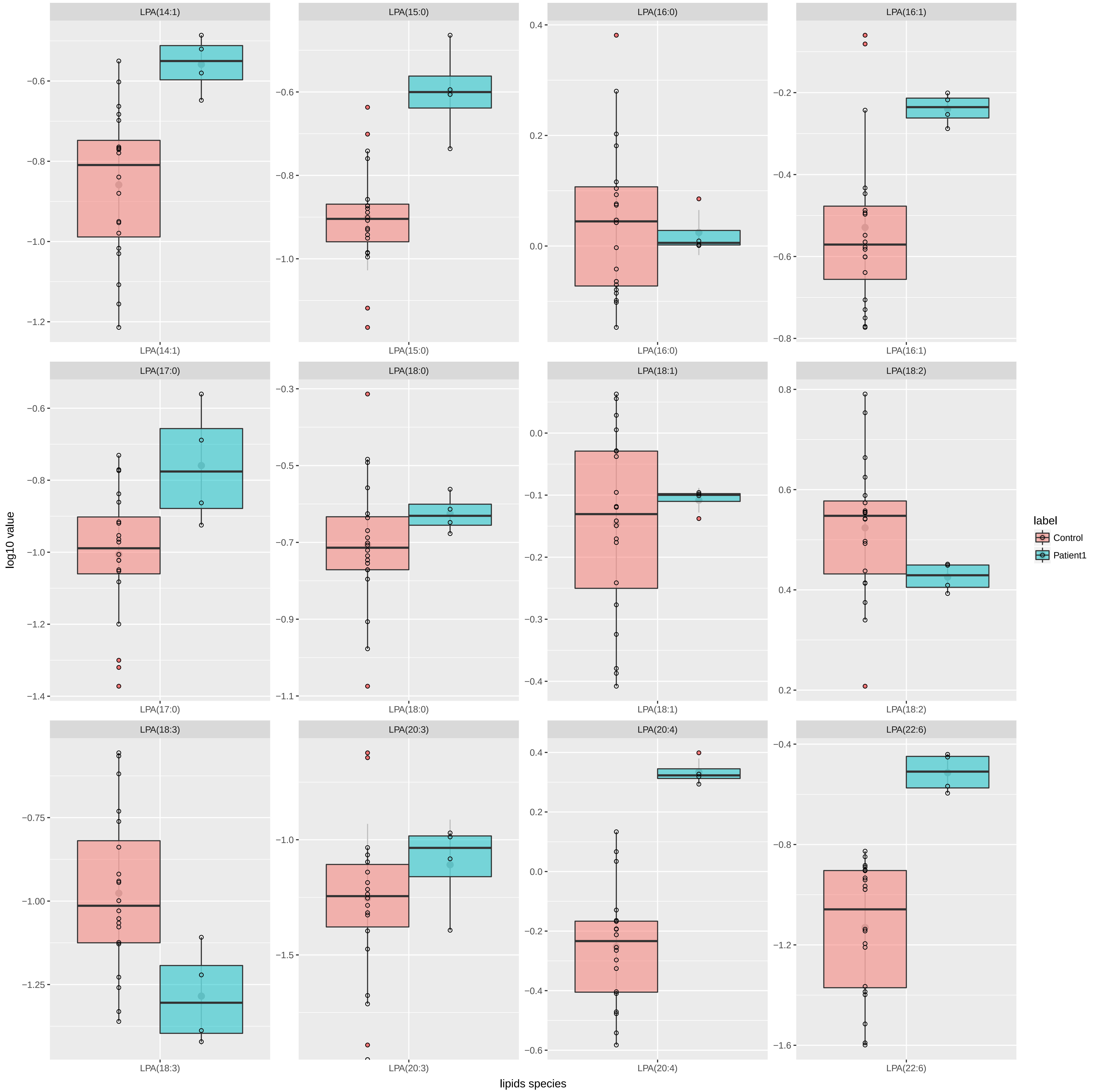

LPA(14:1)
LPA(15:0)
LPA(16:0)
LPA(16:1)
LPA(17:0)
LPA(18:0)
LPA(18:1)
LPA(18:2)
LPA(18:3)
LPA(20:3)
LPA(20:4)
LPA(22:6)
log10 value
lipids species
label
Control
Patient1
